# Structured paediatric endoscopy training: Lessons from curriculum innovation

**DOI:** 10.1111/medu.15717

**Published:** 2025-05-08

**Authors:** Brett J. Hoskins

## 1 WHAT PROBLEMS WERE ADDRESSED?

Traditional paediatric endoscopy training often relies on the apprentice model, where skill acquisition is informal and dependent on exposure during patient care. This unstructured approach can lead to significant variability in fellows' technical proficiency and comfort, especially with therapeutic endoscopic procedures. Despite increasing recognition of simulation and competency‐based frameworks in medical education, these methods remain underutilised in many paediatric programmes. There was a need for an intentionally formalised curriculum to address any disparities in training, ensure consistent skill development and prepare fellows to become independent endoscopists.

## 2 WHAT WAS TRIED?

A structured curriculum was implemented for six paediatric gastroenterology fellows over 1 year, combining simulation, targeted didactics and advanced procedural exposure. Key components included:

1. *Restructuring introductory training*: The first‐year endoscopy month curriculum featured foundational lectures, hands‐on simulation with the Thompson Endoscopic Skills Trainer® (EndoSim, LLC, Bolton, MA, USA), consistent educator teams to standardise instruction and an ‘endoscopy passport’ to track progress.
2. *Advanced therapeutic techniques*: The second‐year endoscopy curriculum emphasised advanced procedures such as polypectomy, stricture dilation, incisional therapy, endoscopic mucosal resection, third space endoscopy, trans‐nasal endoscopy, endoscopic retrograde cholangiopancreatography and endoscopic ultrasound.
3. *Quarterly simulation sessions*: All fellows engaged in routine hands‐on practice with models to reinforce procedural skills.
4. *Educational resources*: Easily accessible endoscopy‐focused video tutorials were introduced to enhance evidence‐based learning.
5. *Peer‐reviewed journal discussions*: All fellows peer‐reviewed literature to deepen their understanding of endoscopy techniques.

Pre‐ and post‐intervention surveys measured perceptions of training adequacy and procedural comfort using a 5‐point Likert scale.

## 3 WHAT LESSONS WERE LEARNED?

The structured curriculum led to significant improvements in fellows' perceptions of formal endoscopy training adequacy (mean score: 2.2 pre‐intervention vs. 4.2 post‐intervention; *p* = 0.003). Perceptions of informal training remained stable (mean: 3.3 vs. 3.7; *p* > 0.05). While fellows' comfort with diagnostic endoscopy was already high, the intervention increased their confidence in therapeutic procedures, though specific skill improvements did not reach statistical significance.

These interventions highlight several key insights into the training and development of skills in therapeutic endoscopy. First, structured learning significantly enhances confidence among trainees. By incorporating formalised training methods, such as simulation and targeted curricula, this approach effectively addresses gaps in traditional training models. While simulation exercises offer valuable hands‐on practice and boost trainee comfort, they also highlight the need for more opportunities to apply these skills in real‐world scenarios. Additionally, the curriculum design plays a crucial role in the learning process. Tools like the ‘endoscopy passport’ are beneficial as they allow fellows to track their progress and facilitate constructive feedback from faculty, enhancing the educational experience.^1^

Despite these successes, limitations included the small cohort size and reliance on self‐reported data, which may not accurately reflect objective competency. The absence of a control group also limited the ability to attribute changes solely to the intervention. Future iterations should expand participant numbers, incorporate faculty evaluations and explore emerging technologies such as virtual reality to supplement traditional simulation. By refining these approaches, programmes can better prepare fellows for the complex demands of paediatric endoscopy.

## AUTHOR CONTRIBUTIONS

**Brett J. Hoskins:** Conceptualization; writing—original draft; methodology; writing—review and editing.

## CONFLICT OF INTEREST STATEMENT

The author is a consultant for Mirum Pharmaceuticals, Inc. There are no relevant conflicts of interest or sources of funding to declare for this manuscript.

## ETHICS STATEMENT

Ethical approval was not required for this study.

## Data Availability

The data that support the findings of this study are available from the corresponding author upon reasonable request.
